# Prenatal exposure to the Great Chinese Famine and mid-age hypertension

**DOI:** 10.1371/journal.pone.0176413

**Published:** 2017-05-12

**Authors:** Lei Wu, Xueqin Feng, Axin He, Yi Ding, Xiuwen Zhou, Zhice Xu

**Affiliations:** 1 Institute for Fetology and Reproductive Medicine Center, First Hospital of Soochow University, Suzhou, Jiangsu, China; 2 Suzhou Industrial Park Centers for Disease Control and Prevention, Suzhou, Jiangsu, China; 3 Center for Perinatal Biology, Loma Linda University, Loma Linda, CA, United States of America; Loma Linda University School of Medicine, UNITED STATES

## Abstract

**Background:**

One of the most terrible famines last century was Great Chinese Famine (GCF) in 1959~1961 when millions of people died from starving. Under-nutrition during famine between the Western and Eastern (Dutch Hungry vs. GCF) was similar, while cardiovascular consequences might not be the same. Addressing such questions may gain new insight into prevention of cardiovascular diseases.

**Methods:**

A retrospective cohort of 18,593 participants aged 43–49 years of old, was from Suzhou, China. Logistic regression model was used to calculate the relative risk (RR) of hypertension and corresponding 95% confidence interval (CI). The multivariate RRs were adjusted for age, plasma glucose, triglyceride, and cholesterol.

**Results:**

The multivariate RRs of systolic and diastolic pressure were not significantly elevated in the rural subgroups, but was higher in the urban population born in the famine (systolic pressure adjust RR 1.382, 95% CI 1.235–1.545, diastolic pressure adjust RR 1.569, 95% CI 1.415–1.740). The risks of hypertension were significantly higher among the urban subjects than that in the rural subgroups (systolic hypertension adjust RR 2.915, 95% CI 2.616–3.249, diastolic hypertension adjust RR 4.568, 95% CI 4.079–5.116). Percentile of optimal diastolic pressure at mid-age was significantly lower in the urban population prenatally exposed to the famine regardless of sexes. However, a similar reduction of percentage of optimal systolic pressure was only seen in the female, not the male population in the urban region.

**Conclusion:**

The data suggest Asian genetic basis was not able to block famine-programmed vascular diseases as that happened in Europe, and the programmed problems due to under-nutrition could be reversed after birth. Protective mechanisms may be related to diet habits before age of 30 years old, which is important contribution to early prevention of hypertension.

## Introduction

Hypertension, one of important cardiovascular diseases, affects billions of people in the world. It is well known that race (genetic) factor plays a role in development of hypertension. For example, the higher incidence was reported in some populations [1], and others such as Chinese were observed relatively low incidence in general [2].

Multiple factors affect development of the essential hypertension. It is no doubt that there is a huge obstacle in prevention and treatments of essential hypertension without sufficient knowledge on causes and mechanisms for the disease. Accumulated evidence in last three decades has made progress in demonstration of programming adult diseases in developmental origins [3]. The hypothesis was proposed that early developmental plasticity affected by environmental insults, e.g. under-nutrition, smoking, cocaine, and so on, could program fetuses for later susceptibility to diseases, including hypertension [4–7]. Substantial literature have shown that maternal under-nutrition is associated with elevation in blood pressure and hypertension in the offspring during adulthood. Among those studies, the famous one studied the subjects borne during “Dutch Hungary” in the World War II, and demonstrated that hypertension was significantly increased in the offspring following prenatal exposure to the famine in Western side of the world [8].

On other side of the globe, one of the most terrible famines took place between the spring of 1959 and the end of 1961, called “Great Chinese Famine” (GCF) [9]. This was listed at top of the most deadly 100 natural disasters of the 20th century. According to China Statistical Yearbook (1984), crop production decreased from 2,000,000 tons (1958) to 1,435,000 tons (1960). Birth rate decreased from 2.922% (1958) to 2.086% (1960), and death rate increased from 1.198% (1958) to 2.543% (1960). All regions in the country suffered the catastrophe. During famine, women conceived and gave birth to babies. The effects of malnutrition during pregnancy on health in adult life have attracted great attention since the theory of adult diseases in fetal origins has been introduced.

Half century later, that disaster left the world a special oriental famine model for studies on diseases in fetal origins. To the best of our knowledge, this was the first study to compare the effects of famine on blood pressure between urban and rural residents and its relation with postnatal diet habits. We selected local individuals, 43~49-year-old, in Suzhou area, a place with both rural and urban populations in Jiangsu province that also was severely affected by GCF. Migration of the residents at those ages in the area hardly happened. According to the records of Suzhou Statistical Bureau, crop production decreased from 25,105 tons (1958) to 4,070 tons (1960). The death rate increased from 8.17‰ (1958) to 15.69‰ (1960). Naturally, under-nutrition caused by famine between the Western and Eastern (Dutch Hungry vs. GCF) was basically similar. Since there are possibilities for differences in genetic or other factors between Europe and Asian populations, cardiovascular consequences after famine between those populations may not the exactly same. Therefore, the present study aimed at the question “Whether and to what extent hypertension development may be affected by prenatal exposure to GCF in Asian population?”. To address such questions may provide new insight into causes and prevention of the diseases.

Besides race or genetic influence on blood pressure, living styles such as diet habits contribute critically to hypertension [10]. It is well known in China that eating habits or types of food consumption were significantly different between urban and rural regions. This provides a unique model for comparisons between the rural and urban areas following *in utero* exposure to GCF. Thus, this study also addressed the question “Whether those people at their 40s in the rural areas suffered less or more from hypertension than the others in the urban following prenatal exposure to GCF?” Answering those questions should be important in gaining novel information for prevention of the disease.

## Methods

### Study cohort

The present study used a retrospective cohort in Suzhou, Jiangsu Province, China. Those who were born between the end of 1959 and 1961 were defined as exposed population, and others (born 1–2 years before 1959 and after autumn of 1962) as non-exposed population. Overall, 20,200 participants aged 43–49 years of old were randomly selected based on a multi-stage sampling method. 18,593 participants (92%) have been followed for at least five years in this cohort. The characteristics of non-participants, such as age, sex, clinical and biochemical characteristics were similar to those who participated in the follow-up survey.

We excluded participants with hypertension, diabetes, cardiovascular diseases, and missing data at baseline. We also excluded participants with familial history of hypertension (either of parents with hypertension), body mass index (weight in kilograms divided by the square of the height in meters) >28, smoking (>1 package daily), or drinking (>50g alcohol daily). After exclusion, the total number of individuals in this study was: n = 18,593.

### Determination of blood pressure

Blood pressure was measured with standard sphygmomanometers from the left arm while the subjects were in a sitting position next to doctors, and recorded as the mean of three successive readings. Both of the doctors and the individuals were not acquainted with analysis of the data. The optimal blood pressure was determined as systolic pressure (SP) ≤120mmHg and diastolic pressure (DP) ≤80mmHg. For this study, hypertension was defined as SP≥140mmHg and/or DP≥90mmHg[11]. Individuals in the present study were those who did not use any medicine or treatment against cardiovascular diseases in order to avoid possible influence from drugs or treatments.

### Covariate measurement

Blood samples were collected in the morning after at least 8 hours of fasting. All plasma and serum samples were frozen at –80°C until testing. Fasting plasma glucose (FPG) was measured using an oxidize enzymatic method. The concentrations of high-density lipoprotein cholesterol (HDL-C), low-density lipoprotein cholesterol (LDL-C), and triglycerides (TG) were assessed enzymatically using an automatic biochemistry analyzer (Hitachi Inc, Tokyo, Japan) and commercial reagents. All analyses were performed by the hospital laboratories.

### Collections of economic and food consumption data

The data for natural growth rate, social economic statistical data in Suzhou area during 1949–1985, and food consumption in Suzhou area were from un-published official records from local Archives. We were kindly allowed to use them in medical/scientific research in this study.

### Statistical analysis

Data were presented as mean ± standard deviation (SD) for continuous variables, and (%) for categorical variables. Logistic regression was used to obtain the crude and adjusted relative risks (RR) of dichotomous outcomes (SP, DP). Data analyses were conducted using the SPSS 21. P value<0.05 were considered statistically significant.

### Ethics

The study was reviewed and approved by the ethics committee board of First Hospital of Soochow University (ER201601197). All participants gave written informed consent before participating in the study.

## Results

### The clinical and biochemical characteristics of the participants

**Fig 1:**
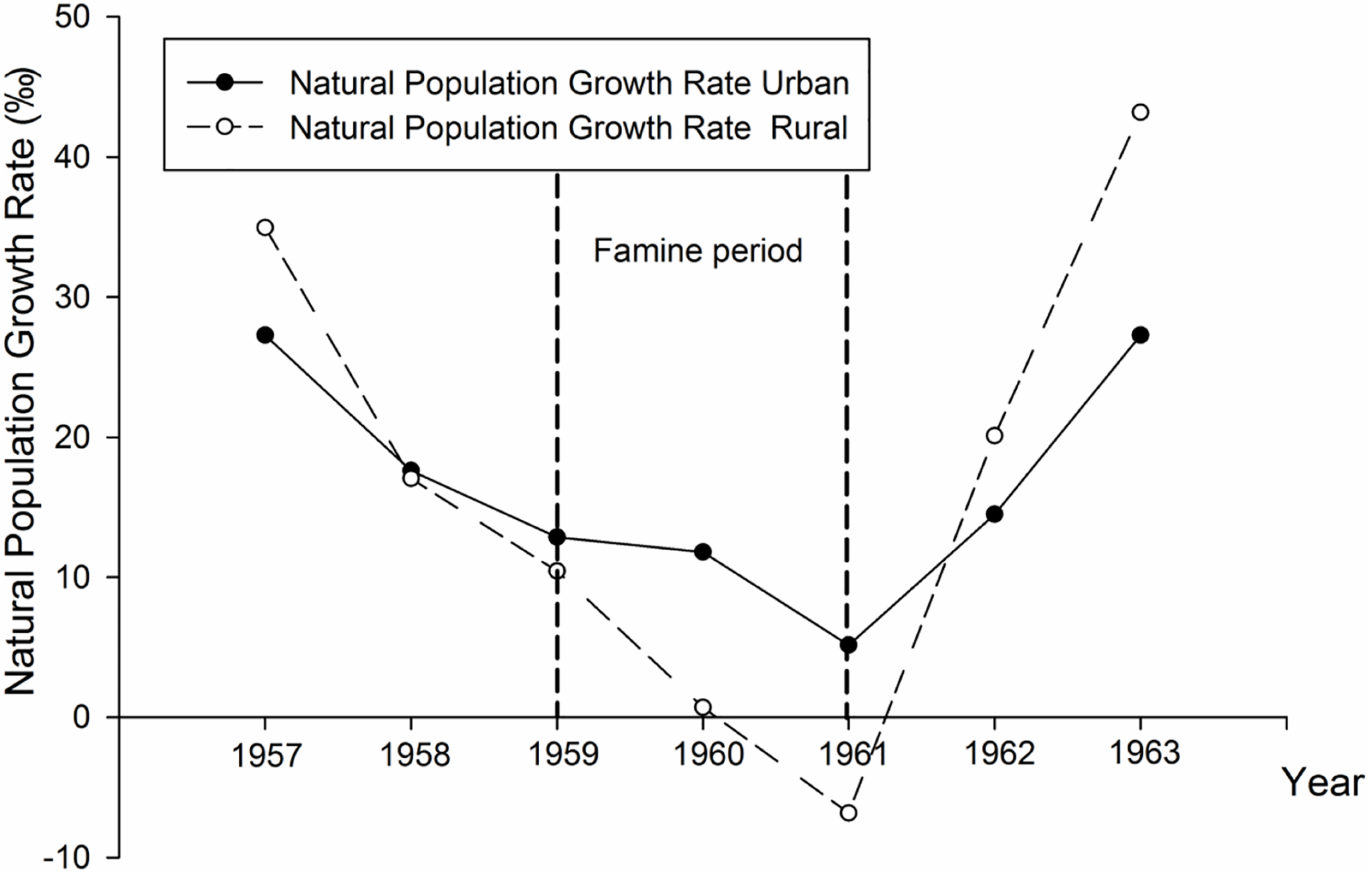
The natural population growth rate in Suzhou area during the time period 1957–1963.

Great Chinese Famine took place between the spring of 1959 and the end of 1961. The natural population growth presents the difference between general natality and mortality. It was 29.29‰ (Urban) and 34.95‰ (Rural) in 1957, whereas in 1961 it dropped to 5.14‰ and -6.85‰ in Suzhou area (Fig 1).

A total of 18,593 participants (9,112 exposed to the famine during fetal stage and 9,481 non-exposed to the famine) were studied; this number included 1,403 subjects with incident systolic hypertension and 1,563 subjects with incident diastolic hypertension by the follow-up investigation. There was no significant difference between the non-exposed and exposed subjects in average age, FPG, TG, HDL-C or LDL-C as shown in Table 1.

**Table 1:** The clinical and biochemical characteristics of the participants.

	Total (N = 18593)	Exposed (n = 9112)	Non-exposed (n = 9481)	*P*-value^#^
**Proportion of Sex (male%)**	53.40%	53.50%	53.30%	0.758^a^
**Age (years)**	46.10±2.15	46.11±2.15	46.09±2.15	0.677^b^
**Fasting Plasma Glucose (mmol/L)**	5.01±1.10	5.03±1.12	5.00±1.07	0.215^b^
**Serum total cholesterol (mmol/L)**	4.67±0.89	4.66±0.89	4.67±0.89	0.768^b^
**Triglyceride (mmol/L)**	1.26 (0.86, 1.91)*	1.25 (0.85, 1.92)*	1.26 (0.86, 1.90)*	0.058
**High-density lipoprotein cholesterol (mmol/L)**	1.44 (1.17, 3.58)*	1.44 (1.17, 3.60)*	1.44 (1.17, 3.54)*	0.312
**Low-density lipoprotein cholesterol (mmol/L)**	3.07±1.69	3.05±0.79	3.09±2.24	0.238^b^

^#^*P* value indicates exposed vs. non-exposed.

* Median and inter-quartile for triglyceride (TG) and high-density lipoprotein (HDL-C) cholesterol.

^a^ Chi-square test for proportion of sex.

^b^ The values shown are the means ± standard deviation for age, fasting plasma glucose (FPG), serum total cholesterol (STC) and low-density lipoprotein cholesterol (LDL-C).

### Hypertension risk of participants in urban and rural area

**Fig 2:**
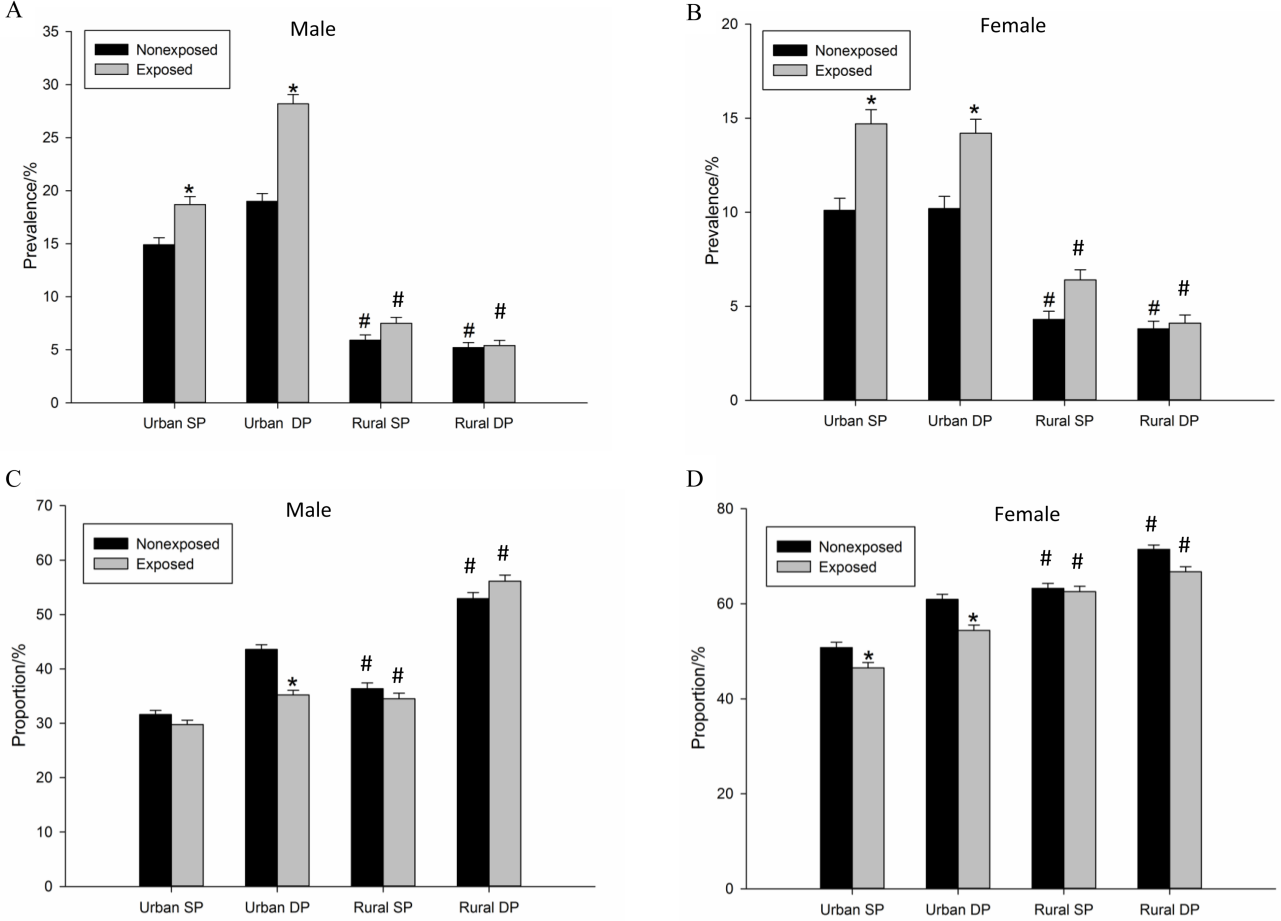
Hypertension prevalence at 43~49-year-old in the male and female following exposure to GCF. **(**A), (C): Male. (B), (D): Female. Non-exposed: un-exposed to the famine; Exposed: prenatally exposed to the famine. SP: systolic pressure; DP: diastolic pressure. (C), (D) The proportion of optimal blood pressure. The optimal blood pressure was determined as ≤120mmHg and ≤80mmHg, and the hypertension were determined as SP≥140mmHg and/or DP≥90mmHg. Total male: n = 9,924; n(urban) = 5,486 [n(exposed) = 2,662, and n(non-exposed) = 2,824]; n(rural) = 4,438 [n(exposed) = 2,212, and n(non-exposed) = 2,226]. Total female: n = 8,669; n(urban) = 4,370 [n(exposed) = 2,182, and n(non-exposed) = 2,188]; n(rural) = 4,299 [n(exposed) = 2,056, and n(non-exposed) = 2,243]. *, p< 0.01(exposed *vs*. non-exposed); #, p< 0.01 (urban *vs*. rural).

Participants were divided into 2 subgroups by region distribution. Fig 2A and 2B showed that prevalence of systolic and diastolic hypertension was significantly higher in the male and female subgroups living in the urban area prenatally exposed to the famine. In the contrast, there was no significant difference of systolic or diastolic hypertension between the non-exposed and exposed subjects in the rural subgroup. Compared with the rural non-exposed population, hypertension prevalence (either systolic or diastolic hypertension) was significantly higher in the non-exposed urban group of both the male and female subjects. The proportion of subjects with optimal DP was significantly decreased in both the male and female subgroups living in the urban area prenatally exposed to GCF (Fig 2C and 2D). In addition, optimal SP in proportion of the female, not the male, was significantly lower in the urban subjects. There was no significant difference in optimal SP and DP in proportion of subjects in the rural subgroups between the non-exposed and exposed subjects.

As shown in Tables 2 and 3, compared with the rural subjects, the multi-adjusted RRs of systolic hypertension and diastolic hypertension were 2.915 (95% CI: 2.616–3.249) and 4.568 (95% CI: 4.079–5.116) for the urban subjects, respectively. Compared with non-exposed subjects in the urban subgroup, the multi-adjusted RRs of systolic hypertension and diastolic hypertension were 1.382 (95% CI: 1.235–1.545) and 1.569 (95% CI: 1.415–1.740) for the exposed subjects, respectively (Tables 2 and 3). However, there was no similar trend in the rural subgroup. Compared with the non-exposed subjects in the rural subgroup, the multi-adjusted RRs of systolic hypertension and diastolic hypertension were 1.182 (95% CI: 0.985–1.419) and 1.058 (95% CI: 0.867–1.291) for the exposed subjects, respectively.

**Table 2:** Relative risks of systolic hypertension of rural and urban residents suffering prenatal famine.

Region	Group	N	Systolic Hypertension			
			Crude *RR*		Adjusted *RR* *	
Rural	Non-exposed	4469	1	1	1	1
	Exposed	4268		1.185 (0.987–1.421)		1.182 (0.985–1.419)
Urban	Non-exposed	5012	2.916 (2.622–3.244)	1	2.915 (2.616–3.249)	1
	Exposed	4844		1.381 (1.234–1.544)		1.382 (1.235–1.545)

* adjustment for age, FPG, TG, HDL-C or LDL-C.

**Table 3:** Relative risks of diastolic hypertension of rural and urban residents following prenatal famine.

Region	Group	N	Diastolic Hypertension			
			Crude *RR*		Adjusted *RR* *	
Rural	Non-exposed	4469	1	1	1	1
	Exposed	4268		1.060 (0.869–1.294)		1.058 (0.867–1.291)
Urban	Non-exposed	5012	4.644 (4.153–5.194)	1	4.568 (4.079–5.116)	1
	Exposed	4844		1.565 (1.412–1.735)		1.569 (1.415–1.740)

* adjustment for age, FPG, TG, HDL-C or LDL-C.

### Hypertension risks between the male and female participants

Participants were divided into 2 subgroups by gender at baseline. As shown in Tables 4 and 5, compared with the female subjects, the multi-adjusted RRs of male systolic hypertension and diastolic hypertension were 1.426 (95% CI: 1.276–1.593) and 1.999 (95% CI: 1.795–2.225), respectively.

Compared with the non-exposed subjects in the male subgroup, the multi-adjusted RRs of systolic hypertension and diastolic hypertension were 1.241 (95% CI: 1.110–1.401) and 1.471 (95% CI: 1.317–1.613) for the exposed subjects (Table 5). Similarly, compared with the non-exposed subjects in the female subgroup, the multi-adjusted RRs of systolic hypertension and diastolic hypertension were 1.459 (95% CI: 1.254–1.696) and 1.358 (95% CI: 1.162–1.586) for the exposed subjects, respectively.

**Table 4:** Relative risks of systolic hypertension in female and male subgroups suffering prenatal famine.

Gender	Group	N	Systolic Hypertension			
			Crude *RR*		Adjusted *RR* *	
Female	Non-exposed	4238	1	1	1	1
	Exposed	4431		1.457 (1.253–1.694)		1.459 (1.254–1.696)
Male	Non-exposed	4874	1.443 (1.310–1.588)	1	1.426 (1.276–1.593)	1
	Exposed	5050		1.238 (1.097–1.397)		1.241 (1.110–1.401)

* adjustment for age, FPG, TG, HDL-C or LDL-C

**Table 5:** Relative risks of diastolic hypertension in female and male subgroups suffering prenatal famine.

Gender	Group	N	Diastolic Hypertension			
			Crude *RR*		Adjusted *RR* *	
Female	Non-exposed	4238	1	1	1	1
	Exposed	4431		1.359 (1.163–1.587)		1.358 (1.162–1.586)
Male	Non-exposed	4874	2.053 (1.868–2.256)	1	1.999 (1.795–2.225)	1
	Exposed	5050		1.466 (1.313–1.636)		1.471 (1.317–1.613)

* adjustment for age, FPG, TG, HDL-C or LDL-C.

### Income and food consumption inequality between the urban and rural areas

**Fig 3:**
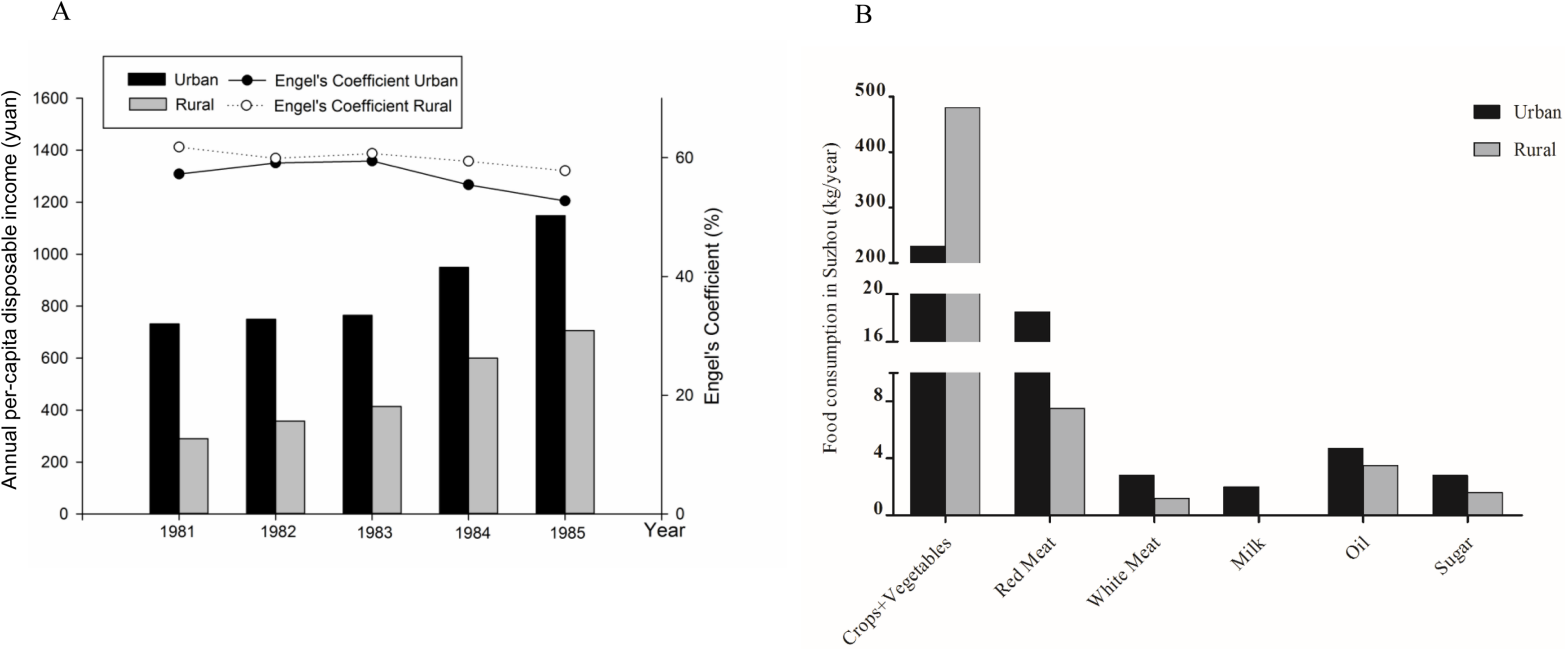
The annual per-capita disposable income and food consumption in Suzhou during the period of 1981–1985. (A) The annual per-capita disposable income and Engel's Coefficient in Suzhou during the period of 1981–1985. (B) The food consumption in Suzhou area in 1981.

The annual per capita disposable income could directly reflect the people’s living standard and consuming capacity. This study considered the income gap between the urban and rural residents. As shown in Fig 3A, the per capita disposable income of local farmers increased from 290 Yuan in 1981 to 706 Yuan in 1985. Ratio by urban-rural income maintained above 1.6:1 from 1981 to 1985. Engel's coefficients of the urban and rural households were stable at 57.25% (1981), 52.73% (1985), 61.8% (1981), and 57.8% (1985), respectively.

Consuming of crops and vegetables of the rural residents was significantly more than that of the urban residents. Whereas, the meat, milk, oil and sugar intake of the rural residents was lower than that of the urban residents.

## Discussion

A significant drop of the natural population growth rate in Suzhou area during the famine period is shown in Fig 1. The great famine from year 1959 to 1961 indeed resulted in serious malnutrition in the area. The exposed subjects aged 43–49 years of old born between 1959 and 1962, whose mothers and themselves as fetuses had suffered from GCF. Compared with the Dutch Hungry, GCF was characterized with two distinctive features: starving period was much longer (3-year vs. 7-month), and affected populations and areas were much larger. Normal human pregnancy lasts nearly 10 months; and no single full-term pregnancy was from Dutch Hungry. Therefore, the Chinese model can be used to study health problems originated from prenatal insults in different exposed time and different geographic regions. During GCF, the natural population growth rate in Suzhou area was decreased from 27.29‰/34.95‰ (1957–1958) to 5.14‰/-6.85‰ (1961) in the urban / rural area (Fig 1).

Numerous studies assessed effects of environmental and genetic factors in development of hypertension, and numerous candidate genes have been investigated, although few have been confirmed [12–14]. The studies on the Dutch model have shown that the Western people prenatally exposed to famine had an increased prevalence of coronary heart disease and hypertension [15]. Following exposure to the Chinese famine, the urban people at mid-age also suffered from a higher prevalence of systolic and diastolic hypertension regardless of genders. Notably, there were significant differences of risks in hypertension in the exposure populations between the urban and rural area in the present study. Compared with the studies showing increased risk for hypertension and other cardiovascular diseases in the Western famine model [16–18], the present study demonstrated two important findings: First, protective mechanisms related to race/genetic factors may be unable to block hypertension development from prenatal malnutrition; Second, cardiovascular diseases programmed in fetal origins could be preventive or reversed after birth. The significance from the first finding is adding new information on that prenatal insult-increased risks for hypertension can occur in Europe as well in Asia, no matter how different genetic basis could be between the Western and Eastern. The second finding is particular important because it indicates that the prenatal factor-increased cardiovascular problems could be reversed during postnatal life, providing new information for prevention of hypertension or other cardiovascular diseases.

It is widely accepted that SP/ DP not over 120/80 mmHg is viewed as optimal blood pressure [11]. The present study demonstrated that, both in men and women, the proportion of the optimal blood pressure (either SP or DP) in the subjects, whose mothers suffered a great famine in pregnancy, were decreased (Fig 2). It means that the effect of the famine accelerates the increase of the blood pressure in the population, which could elevate the incidence of hypertension as getting older. In addition, it also supported the finding that the population who underwent a fetal under-nutrition would be more susceptive to hypertension. Notably, percentile of optimal DP at mid-age was significantly lower in the urban population prenatally exposed to the famine regardless of sexes. However, a similar reduction of percentage of optimal SP was only seen in the female, not the male population in the urban region. In study of prenatal influence following Dutch Hungry, researchers found gender differences in behavioral, neural, and mental development, as well as related diseases [19–22]. In the present study, although increased prevalence of systolic hypertension was observed in the male urban population, percentile of optimal SP was not affected in the same group, indicating that gender factor either at genetic or hormonal levels may play a role in keeping a good blood pressure.

Another interesting and important finding in the present study was that the rural people prenatally exposed to GCF was not significantly associated with higher risk of hypertension as that in their neighbor urban population (Tables 2 and 3). There were significant differences in risks of hypertension between the urban and rural mid-age populations following prenatal exposure to the famine, indicating that the blood pressure of the urban was more likely to be affected by the risk factor of the prenatal insult than the rural people. Not like what observed in the urban people, neither prevalence of hypertension nor optimal blood pressure population of both the male and female in the rural areas was significantly changed by the famine during the prenatal period, suggesting protective mechanisms existed in the countryside for its mid-age residents against the impact in fetal origins after GCF. In determination of possible protective mechanisms, we carefully excluded number of factors such as smoking, drinking, using of medicine, high body mass index, and adjust for age, plasma glucose, serum total cholesterol triglyceride, high-density lipoprotein cholesterol and low-density lipoprotein cholesterol, which may be linked to an increase of blood pressure. Then we focused on diet habits between the urban and rural areas, since it is well known, besides genetic influence, eating habits or diet styles play vital roles in cardiovascular health and diseases, including hypertension [23, 24].

Although it is also well known in China that eating habits or types of food consumption were significantly different in general, between urban and rural regions, mainly due to huge gap of economic differences before 1990 (after that time, the economic differences between Suzhou urban and rural regions was significantly changed), the data in reliable number are always on a solid basis for any scientific explanation or conclusion. Thanks the local Statistical Bureau that did data collecting and analysis before, and kept the un-published records for decades. Those data showed, for the exposed population before age of 30 years old (before 1990) after birth, that consuming of crops and vegetable was significantly higher in the rural area of Suzhou than that in the urban, while consuming of meat (both red and white meat), sugar, milk with fat, and eatable oil were obviously lower in the rural areas than those in the urban regions of Suzhou (Fig 3). Although living conditions in the agricultural areas was constantly improved with the steady increase of income, the urban-rural income ratio was 2.5: 1.0–1.6: 1.0 from 1981 to 1985 (Fig 3) as an example. The “Engel's coefficient”, which indicates the ratio between the expenses of food and other consumption remained stable in the urban and rural households between 1980s and 1990s (Fig 3). Based on those economic and food consuming style data in the rural-urban area, the retrospective analysis of the famine data strongly suggest that the significantly reduced risks in hypertension in the mid-aged rural exposed group might be benefited from their eating habits before age of 30 years old. In other words, even extent of prenatal malnutrition by the famine was much terrible in the rural area than the urban, which can be reflected by the natural population growth rate and other data recorded 50 years ago, cardiovascular risks due to prenatal origins were significantly lowered in the rural region compared to the urban area in Suzhou. We realized that development of hypertension is mediated rather by complex mechanisms. Many factors that may contribute to the disease were carefully excluded in this study. The huge differences in food consumption between the rural and urban area before 1990 could not be neglected and attracted great attention. The nutritional status during adolescence is crucial for adulthood blood pressure in both females and males [25]. A previous cohort study in US suggested that adopting a low-fat diet too late in life may not be able to reverse the risk of cardiovascular diseases [26]. The novel finding in the present study provided important information on a reversible relationship between prenatal malnutrition-programmed cardiovascular risks and postnatal dieting styles adapted before age of 30 years old. The present study was the first in demonstration that hypertension development at mid-age following prenatal malnutrition could be preventive, and possible protective secrets may be linked to the diets after birth and before age of 30 years old. This adds novel and important information to the hypothesis of adult diseases in fetal origins [4, 27, 28].

The present study also showed the risks of hypertension were elevated for the exposed group both in the male and female cohort, while the risks were higher in the male cohort than that of the female cohort (Tables 4 and 5). Notably, the involved subjects were at their mid-age, differences in sex hormones between the male and female for that age should be considered, as a number of studies have demonstrated that the female hormones play critical roles in prevention of cardiovascular diseases before menopause [29]. Details of the underlying mechanisms are worth for further investigations. The present study demonstrated that prenatal malnutrition-initiated cardiovascular risks could be reversed by postnatal nutrition (daily food intake), the age period before 30-year-old was important for health in the mid-age, and probably for the old too. Considering that numerous studies have shown that prenatal malnutrition is also linked to other chronic major diseases or conditions besides hypertension, including diabetes, obesity, mental illness, etc.[30, 31], the finding in the present study also offered new insight into early prevention of those chronic adult diseases in fetal origins.

In conclusion, the present study revealed some similarities and differences in development of hypertension in fetal origins between the Western and Eastern famine models, providing novel information on prevalence of hypertension following prenatal exposure to GCF. Interestingly and importantly, the data from the rural areas together with those previous un-published food consuming results strongly suggest that prenatal insult-programmed cardiovascular diseases, at least risks for development of the diseases, could be reduced or reversed in postnatal life, especially before age of 30 years old, by changing eating habits simply.

## References

[1] Baskar V, Kamalakannan D, Holland MR, Singh BM. Does ethnic origin have an independent impact on hypertension and diabetic complications? Diabetes Obesity & Metabolism. 2006;8(2):214–9. 10.1111/j.1463-1326.2005.00485.x 16448526

[2] Wu X, Duan X, Gu D, Hao J, Tao S, Fan D. Prevalence of hypertension and its trends in Chinese population. International Journal of Cardiology. 1995;52(1):39–44. 8707434 10.1016/0167-5273(95)02443-z

[3] Barker DJP. The developmental origins of adult disease. Current Opinion in Pediatrics. 2004;23(6):588S–95S. 10.1080/07315724.2004.10719428 15640511

[4] Barker DJ. Fetal and infant origins of adult disease. Monatsschrift Kinderheilkunde. 2001;149(1):S2–S6.

[5] Edwards LJ, Coulter CL, Symonds ME, Mcmillen IC. Prenatal Undernutrition, Glucocorticoids And The Programming Of Adult Hypertension. Clinical & Experimental Pharmacology & Physiology. 2001;28(11):938–41. 11703401 10.1046/j.1440-1681.2001.03553.x

[6] Somm E, Schwitzgebel VM, Vauthay DM, Aubert ML, Hüppi PS. Prenatal nicotine exposure and the programming of metabolic and cardiovascular disorders. Molecular & Cellular Endocrinology. 2009;304(s 1–2):69–77. 19433250 10.1016/j.mce.2009.02.026

[7] Daliao X, Xiaohui H, Zhice X, Shumei Y, Lubo Z. Prenatal cocaine exposure differentially causes vascular dysfunction in adult offspring. Hypertension. 2009;53(6):937–43. 10.1161/HYPERTENSIONAHA.108.121830 19380615 PMC2698595

[8] Barker D. Mothers, Babies and Health in Later Life. Public Health. 1999;113(5).

[9] Smil V. China's great famine: 40 years later. Bmj British Medical Journal. 1999;319(7225):1619–21. 10600969 10.1136/bmj.319.7225.1619 PMC1127087

[10] Forman JP, Stampfer MJ, Curhan GC. Diet and lifestyle risk factors associated with incident hypertension in women. Jama the Journal of the American Medical Association. 2009;302(4):401–11. 10.1001/jama.2009.1060 19622819 PMC2803081

[11] Sethi AA, Nordestgaard BG, Tybjærg-Hansen A. Angiotensinogen gene polymorphism, plasma angiotensinogen, and risk of hypertension and ischemic heart disease a meta-analysis. Arteriosclerosis, thrombosis, and vascular biology. 2003;23(7):1269–75. 10.1161/01.ATV.0000079007.40884.5C 12805070

[12] Lohmueller KE, Wong LJ, Mauney MM, Jiang L, Felder RA, Jose PA, et al Patterns of genetic variation in the hypertension candidate gene GRK4: ethnic variation and haplotype structure. Annals of human genetics. 2006;70(Pt 1):27–41. 10.1111/j.1529-8817.2005.00197.x 16441255

[13] Cappuccio FP. Ethnicity and cardiovascular risk: variations in people of African ancestry and South Asian origin. Journal of human hypertension. 1997;11(9):571–6. 9364274 10.1038/sj.jhh.1000516

[14] Roseboom TJ, Van Der Meulen JH, Ravelli AC, Osmond C, Barker DJ, Bleker OP. Perceived health of adults after prenatal exposure to the Dutch famine. Paediatric and Perinatal Epidemiology. 2003;17(4):391–7. 14629322 10.1046/j.1365-3016.2003.00516.x

[15] Stein AD, Zybert PA, Van dP-d BK, Lumey LH. Exposure to famine during gestation, size at birth, and blood pressure at age 59 y: evidence from the Dutch Famine. European Journal of Epidemiology. 2006;21(10):759–65. 10.1007/s10654-006-9065-2 17082900

[16] Painter RC, de Rooij SR, Bossuyt PM, Phillips DI, Osmond C, Barker DJ, et al Blood pressure response to psychological stressors in adults after prenatal exposure to the Dutch famine. Journal of hypertension. 2006;24(9):1771–8. 10.1097/01.hjh.0000242401.45591.e7 16915026

[17] Roseboom T, de Rooij S, Painter R. The Dutch famine and its long-term consequences for adult health. Early human development. 2006;82(8):485–91. 10.1016/j.earlhumdev.2006.07.001 16876341

[18] Liu LS. [2010 Chinese guidelines for the management of hypertension]. Chinese Journal of Hypertension. 2011;39(7):579–615. 22088239

[19] Brown AS, van Os J, Driessens C, Hoek HW, Susser ES. Further evidence of relation between prenatal famine and major affective disorder. The American journal of psychiatry. 2000;157(2):190–5. 10.1176/appi.ajp.157.2.190 10671386

[20] Brown AS, Susser ES. Sex differences in prevalence of congenital neural defects after periconceptional famine exposure. Epidemiology (Cambridge, Mass). 1997;8(1):55–8. 10.1097/00001648-199701000-00009 9116096

[21] Brown AS, Susser ES, Lin SP, Neugebauer R, Gorman JM. Increased risk of affective disorders in males after second trimester prenatal exposure to the Dutch hunger winter of 1944–45. The British journal of psychiatry: the journal of mental science. 1995;166(5):601–6. 7620744 10.1192/bjp.166.5.601

[22] Franzek EJ, Sprangers N, Janssens A, Van Duijn CM, Van De Wetering BJ. Prenatal exposure to the 1944–45 Dutch ‘hunger winter’and addiction later in life. Addiction. 2008;103(3):433–8. 10.1111/j.1360-0443.2007.02084.x 18190668

[23] Irwin B. Nutrition and cardiovascular health. Pediatric Annals. 2014;67(9):738–47. 10.1016/j.rec.2014.05.003 25172070

[24] Truswell, Stewart A. DIET AND HYPERTENSION. British Medical Journal. 1985;291(6488):125–7. 3926080 10.1136/bmj.291.6488.125 PMC1416247

[25] Krupp D, Shi L, Egert S, Wudy SA, Remer T. Prospective relevance of fruit and vegetable consumption and salt intake during adolescence for blood pressure in young adulthood. European journal of nutrition. 2015;54(8):1269–79. 10.1007/s00394-014-0804-y 25410750

[26] Howard BV, Van Horn L, Hsia J, Manson JE, Stefanick ML, Wassertheil-Smoller A, et al Low-fat dietary pattern and risk of cardiovascular disease: The women’s health initiative randomized controlled dietary modification trial. JAMA. 2006;295(6):655–66. 10.1001/jama.295.6.655 16467234

[27] Barker D. The developmental origins of adult disease. Journal of the American College of Nutrition. 2004;23(sup6):588S–95S. 15640511 10.1080/07315724.2004.10719428

[28] Barker DJP. Mothers, babies, and health in later life: Elsevier Health Sciences; 1998.

[29] Vitale C, Mendelsohn ME, Rosano GM. Gender differences in the cardiovascular effect of sex hormones. Nature Reviews Cardiology. 2009;6(8):532–42. 10.1038/nrcardio.2009.105 19564884

[30] de Rooij SR, Painter RC, Phillips DI, Osmond C, Michels RP, Godsland IF, et al Impaired insulin secretion after prenatal exposure to the Dutch famine. Diabetes Care. 2006;29(8):1897–901. 10.2337/dc06-0460 16873799

[31] Patrick B, David B, Timothy CB, Debal D, Bruno DU, Foley RA, et al Developmental plasticity and human health. Nature. 2004;430(6998):419–21. 10.1038/nature02725 15269759

